# Supplementary material for: Healthy-unhealthy animal detection using semi-supervised generative adversarial network
Source: PeerJ Comput Sci. 2023 Feb 15;9:e1250. doi: 10.7717/peerj-cs.1250 (PMC10280485; doi:10.7717/peerj-cs.1250)
Supplement: Supplemental Information 1 [file peerj-cs-09-1250-s001.docx]

**Code Link**

<https://github.com/shubh0125/Healthy-Unhealthy-Animal-Classification> (<https://doi.org/10.5281/zenodo.7475604>)
